# PHAROS 2.0—A PHysical Assistant RObot System Improved

**DOI:** 10.3390/s19204531

**Published:** 2019-10-18

**Authors:** Ester Martinez-Martin, Angelo Costa, Miguel Cazorla

**Affiliations:** 1 RoViT, University of Alicante, 03690 San Vicente del Raspeig (Alicante), Spain; miguel.cazorla@ua.es; 2 ALGORITMI Center, University of Minho, 4704-553 Braga, Portugal; acosta@di.uminho.pt

**Keywords:** assistive robotics, active ageing, decision support system, cognitive assistant, deep learning

## Abstract

There are great physical and cognitive benefits for older adults who are engaged in active aging, a process that should involve daily exercise. In our previous work on the PHysical Assistant RObot System (PHAROS), we developed a system that proposed and monitored physical activities. The system used a social robot to analyse, by means of computer vision, the exercise a person was doing. Then, a recommender system analysed the exercise performed and indicated what exercise to perform next. However, the system needed certain improvements. On the one hand, the vision system captured the movement of the person and indicated whether the exercise had been done correctly or not. On the other hand, the recommender system was based purely on a ranking system that did not take into account temporal evolution and preferences. In this work, we propose an evolution of PHAROS, PHAROS 2.0, incorporating improvements in both of the previously mentioned aspects. In the motion capture aspect, we are now able to indicate the degree of completeness of each exercise, identifying the part that has not been done correctly, and a real-time performance correction. In this way, the recommender system receives a greater amount of information and so can more accurately indicate the exercise to be performed. In terms of the recommender system, an algorithm was developed to weigh the performance, temporal evolution and preferences, providing a more accurate recommendation, as well as expanding the recommendation to a batch of exercises, instead of just one.

## 1. Introduction

The world as we know it is changing rapidly, with social and economic issues that were unforeseen a few years ago now having a major impact. The rapid increase in life expectancy has revealed a direct association between cognitive/physical problems and an individual’s age. For people aged 23 over 65 years the likelihood of developing Alzheimer’s disease doubles every five years, increasing steadily with age [1,2]. By 2050, the number of people with severe cognitive disabilities is predicted to be three times larger than today (about 152 million worldwide) [1,3]. In addition, there is an exponential increase in physical problems that affect the performance of activities for daily living (ADLs), caused by common physical limitations that come with age or as a result of more severe acquired medical problems as Parkinson’s disease or osteoporosis.

While medical advances are only currently establishing markers for early detection of these medical issues, diagnosis and treatment are mostly well established. One way to address this problem is by introducing activities that stimulate ageing adults’ brains and/or bodies [3,4]. The World Health Organisation states that “*globally, 23% of adults and 81% of school-going adolescents are not active enough*” and that “*physical activity reduces the risk of coronary heart disease and stroke, diabetes, hypertension, various types of cancer including colon cancer and breast cancer, as well as depression. Physical activity is also fundamental to energy balance and weight control*” [4]. Older adults with poor mobility should do physical activities to enhance balance and prevent falls three or more days per week for at least 150 min, if they are able. Otherwise, they should perform personalised activities that allow them to be as physically active as possible. For instance, if they are wheelchair-bound, they can perform upper body exercises.

Studies show that physical exercise has a highly positive impact on overall health status (even cognitively) [5]. Apart from the usual benefits of regular physical activity, there are others that could greatly improve an older adult’s life, such as reducing the risk of high blood pressure, stroke, diabetes, cancer or depression [4,5]. Furthermore, specific exercises designed to improve balance may result in a lower risk of falling and of hip or vertebral fractures, which is critical to this population.

The *active ageing concept* focuses on keeping older adults active through engaging them in social and individual activities while smoothing the transition from working life (full-time employment) to retired life [6,7]. The issue with the abrupt working/non-working transition is that people find themselves with substantial free time and, more often than not, they spiral down into a dull and inactive life, which, as stated before, leads to health problems. For this reason, a large number of social associations have been created to promote active ageing and create networks between elderly people, proposing social activities and connecting them with companies where they can do volunteer work.

Through their national healthcare systems, a number of countries have invested in promoting active ageing in the form of media platforms for the elderly community (one of the most affected populations) with physical exercises that they can perform at their homes. These exercises are specifically designed to attend to their limitations and to improve (or prevent the decline of) their health. For instance, the British National Health Service (NHS) [8] has designed sets of physical exercises that older people (including those with mild cognitive/physical disabilities) may perform at home to improve their strength, flexibility and balance.

Apart from the physical gains, physical exercise has a positive cognitive impact [9]. It is well known there is a high prevalence of depression in older people and that it is harder to treat at later stages of life [10,11,12]. While the causes for depression are unknown (and do not follow a range of well-established risk factors), it is recognised that physical exercise is one of the possible treatments for this issue, being a low-cost and all-natural solution [10,11,12].

The issue with exercise guidelines like those in Reference [8] is that, without assistance, older adults (given their medical issues) may perform them poorly or even suffer an injury. Two of the most widely adopted solutions are hiring a homecare assistant or going to a specialised gym but these are costly and, in addition, the second option requires travelling, which may not always be possible. Currently, families and elderly people cannot make large financial investments and governments rely on outdated social security models that cannot sustain the sheer number of people that need this type of assistance [13]. As stated in Reference [13], this trend is growing and there are currently no foreseeable solutions. An alternative to this is technological advances like social assistive robots or cognitive assistants. They can be used to monitor and advise older adults in performing tasks or exercises, keeping them company and helping in emergency situations like falls.

In this paper, we present the next generation of PHAROS [14]—PHAROS 2.0. Its goal is to monitor and assist elderly people in their homes, being especially designed for suggesting and monitoring physical exercise performance. This version presents updates in terms of motion capture and the recommender system. The motion capture is able to indicate the degree of completeness of each exercise, identifying which part has not been done correctly and offering real-time performance correction. The recommender system now has an algorithm that weighs the performance, temporal evolution and preferences, to provide a more accurate recommendation, as well as to expand the recommendation to a batch of exercises, instead of just one.

This paper is structured as follows—Section 2 reviews the literature in matters of technological solutions for healthcare. PHAROS 2.0 is presented in Section 3. The new and improved physical exercise recogniser and recommender are described in Section 4 and Section 5, respectively. Experimental results are shown and discussed in Section 6. Finally, some conclusions and future work are presented in Section 7.

## 2. State of the Art

Extensive research to develop technologies complementing and strengthening healthcare has arisen from the continuously growing rate of the elderly and patients with chronic illness. In particular, physical activity is a highly demanding area due to its wide range of applications and benefits [15]. In addition, the difficulties in accessing the services, the intensity and duration demand technological solutions.

As a first step, technological solutions have been developed to assist therapists and healthcare professionals in providing effective, repetitive training and quantitative evaluation of the user’s progress in the hospital environment [16,17,18,19,20,21]. For this, several levels and settings allow the physical guidelines to be tailored for each user in certain ways. Nevertheless, the challenge lies in continuing this therapy outside the hospital setting.

In this regard, a solution could be the use of the capabilities of the *Internet of Things* (IoT) for healthcare. To this end, smart sensors (e.g., *Fitbit* [22]) monitor users and provide clinicians with the required information about the user. Nevertheless, these technologies may infringe upon the user’s privacy since they might provide their details and activity to third parties, making users reluctant to utilise them [23]. Moreover, there are key strategic problems directly related to the rehabilitation quality, such as the management of resources, knowledge and big data, which are still unresolved.

Alternatively, *virtual* therapists have been used as a complementary tool to in-person rehabilitation treatment. In fact, these tools are used in both hospital and in-home settings, in such a way that in-clinic supervised learning facilitates at-home therapy [24]. This is the case of Toyra [25], a virtual reality environment for occupational rehabilitation. Basically, its workflow could be described as monitoring the patient’s movements, transferring them to a virtual world and sending them to the central server for storage in a centralised database. Note that worn body sensors are required to properly measure the user’s movements, considerably limiting its use. With the aim of overcoming this inconvenience, Jintronix presented the *Jintronix Rehabilitation System* (JRS) [26], a virtual-reality platform for physical rehabilitation. In this case, the patient’s recovery is visually analysed session-by-session in terms of movement accuracy, session duration, reaction time and improvement with respect to their previous performances. This data is provided to a therapist who adapts the activity program in view of the user’s needs.

Going a step further, assistive robots may offer a useful solution. This emerging topic has resulted in several proposals. The aim of the first developments was to show and describe the physical exercises but no monitoring was performed (e.g., Reference [27]). In the next step, a training stage with a therapist was required to properly evaluate the user’s performance [28,29]. Recent advances in robotics technologies have led to more autonomous robots. For instance, the European-funded project ENRICHME (ENabling Robot and assisted living environment for Independent Care and Health Monitoring of the Elderly) [30] developed an interactive mobile robot in an assisted living environment. Among its typical services, this robot includes an exercise reminder and monitoring, together with acoustic and graphic exercise description, although no feedback about the user’s performance is provided. Following this line, our previous work was also developed [14].

Nevertheless, despite the wide research on this topic, two main shortcomings should be addressed. The first corresponds to exercise evaluation. Specifically, the *accuracy* measurement is obtained from comparing the user’s movement and the expected one, always assuming that the exercise performed corresponds to that required. Therefore, the feedback is based on this data and no reaction or adaptation takes place when the user performs a different exercise, decides to leave the session or any other situation occurs. This issue has been considered and integrated in the **PHAROS 2.0** system, as explained in the following sections.

Furthermore, the exercise workout is rigid in the sense that a therapist or health professional sets the user’s daily workout. The daily repetition of this workout may give rise to boredom for the user. As a solution, **PHAROS 2.0** has extended its physical exercises with the purpose of engaging the user in the active ageing process. Thus, the recommender system chooses a different physical exercise from a wide range of exercises aimed at the same problem, based on several aspects, such as the user’s likes (see Section 5).

Thus, the main contributions of this paper can be summarised as follows:

- The action dataset was expanded to include incorrect performances of the physical exercises considered. That is, an analysis of physical exercises revealed a series of common errors when doing them. These errors were recorded and added to the action dataset to be taken into account for exercise monitoring
- A new CNN-RNN architecture for exercise recogniser is used (a comparative study showed that the new architecture provides better results than the one used in PHAROS [24])
- In this version, PHAROS 2.0 gives feedback to the user during the exercise. That is, the inclusion of erroneous poses allows PHAROS 2.0 to detect these situations and inform the user so that the pose can be solved before ending the exercise
- A different strategy for the recommender is proposed. The previous version degraded over time, while in this new version enriched information from the exercise recogniser results in a more tailored workout. In addition, this new strategy leads to more variations of exercises, which increases the user’s engagement in following the therapist’s recommendations
- Medical personnel are provided with more detailed information about the user performance in terms of errors made during each physical exercise
- The system was tested and validated in a real care home. This pilot study allowed us to evaluate the performance of PHAROS 2.0 in a real environment and to obtain the future user’s feedback

## 3. PHAROS 2.0

**Figure 1:**
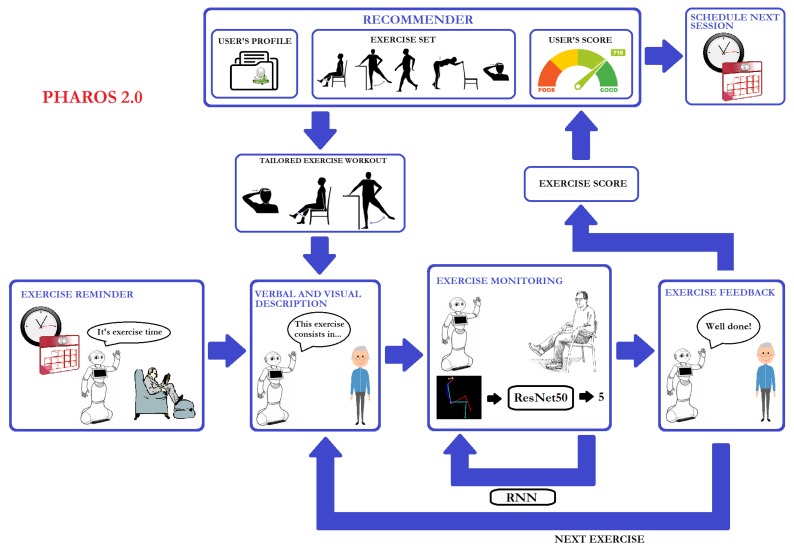
Workflow of PHAROS 2.0.

As illustrated in Figure 1, the PHAROS workflow can be summarised as follows: the interaction starts with a reminder of the daily session of physical exercise. When the user is ready, a tailored workout is generated. Each physical exercise is visually and verbally described and then the user is monitored to be assisted and evaluated so that rich information can be obtained and delivered to the medical personnel. To this end, the following modules were implemented:

- *An exercise timetable*: in charge of reminding the user of the scheduled exercise session
- *A recommender*: its goal is to tailor exercise programs in order to engage the users in the active ageing process
- *An exercise descriptor*: simulates an exercise assistant by visually and verbally describing each physical exercise to be done
- *An exercise recogniser*: with the purpose of providing feedback to the user as well as relevant information to the medical personnel. This module processes visual input to properly identify the exercise done by the user and evaluate their performance
- *A user’s feedback system*: this module is in its early development and, consequently, the feedback provided is highly limited

Note that, at this stage, the human-robot interaction was purposefully limited to reminders, exercise descriptions and feedback (no social component has been included). However, in the near future, we are planning to integrate some ways to properly interact with PHAROS [31,32,33,34,35,36].

## 4. Physical Exercise Recogniser

According to Moeslund and Granum [37] and Poppe [38], an action is described as a diverse range of movements defined at limb level. From this starting point, a physical exercise is considered an action and, consequently, is described as a sequence of limb movements. However, a human representation in terms of limbs or articulations is necessary to achieve this description.

**Figure 2:**
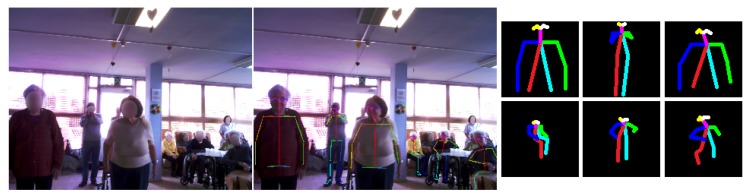
Human representation in terms of limbs by using Openpose: (**left**) Original image taken by the Pepper robot; (**middle**) Openpose output as body keypoint coordinates; (**right**) The human skeleton colour-coded to properly distinguish each limb (one per person).

Thus, the first step is to obtain this human representation from the robot visual system. This problem has been widely studied due to its wide variety of applications, which range from activity recognition to human computer interaction. The main goal, then, of these approaches is to estimate a parameterised 2D or 3D human body model from visual data. In this context, deep learning has led to great advances in 2D and 3D pose estimation from single video sources [39,40,41]. In our study, Openpose [42,43] is used. This open-source library iteratively predicts affinity fields encoding part-to-part association and detection confidence maps to properly detect multi-person keypoints in real-time. A total of 25 human body keypoints are obtained from each person (see Figure 2). As can be observed, this articulation information is translated into a body skeleton that is colour-coded to properly distinguish each limb. Note that the size of the skeleton depends on the individual’s dimensions. Therefore, all the skeletons are isolated, cropped and resized to 24×24 RGB images, aiming to normalise the input to our system.

**Figure 3:**
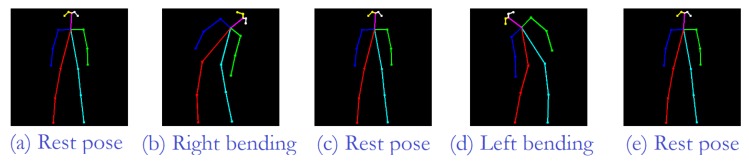
Five-pose sequence showing the exercise sideways bend in terms of limb positions. So, the first human pose corresponds to the rest pose. The next pose shows the pose when the right arm has been slid down towards the right foot. The rest pose is the following step. Then, the left arm has been slid down towards the left foot. The finally pose corresponds to the rest pose.

Once a human representation is obtained, the next step is to describe any physical exercise considered as a sequence of movements at the limb level. For this, all the primitive human poses are identified. So, for instance, an exercise consisting of bending sideways is described as a five-pose sequence (see Figure 3): (1) the rest pose, where the person is standing upright with their feet slightly apart and arms by their sides; (2) the right arm is slid down towards the right foot; (3) the person goes back to the rest pose; (4) the left side is now working, sliding down the left arm towards the left foot; and, (5) the last pose corresponds again to the rest pose.

**Figure 4:**
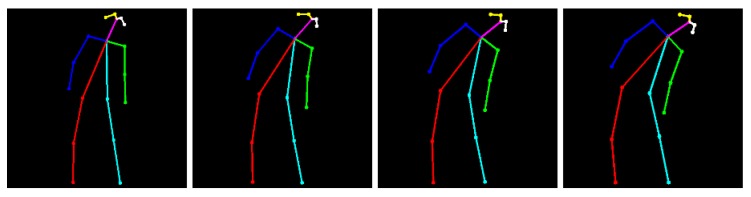
Some samples of different limb positions corresponding to the same human pose: right bend.

It should be noted that only the *key* limb positions are considered despite the complete movement including several intermediate limb positions, as depicted in Figure 4. Thus, each primitive pose is defined as a range of limb positions to include these intermediate poses. This fact, apart from covering the whole exercise, also provides the system with the ability to monitor people with reduced mobility who, far from achieving the *ideal* primitive pose, reach their limb limit at any previous (intermediate) pose. Thus, on occasions, this reduction in mobility may result in partial performance. This partial performance is included in the system by means of exercise sub-sequences. Note that these *partial* definitions receive a score according to the missed primitive poses, as described in our previous work [14].

**Figure 5:**
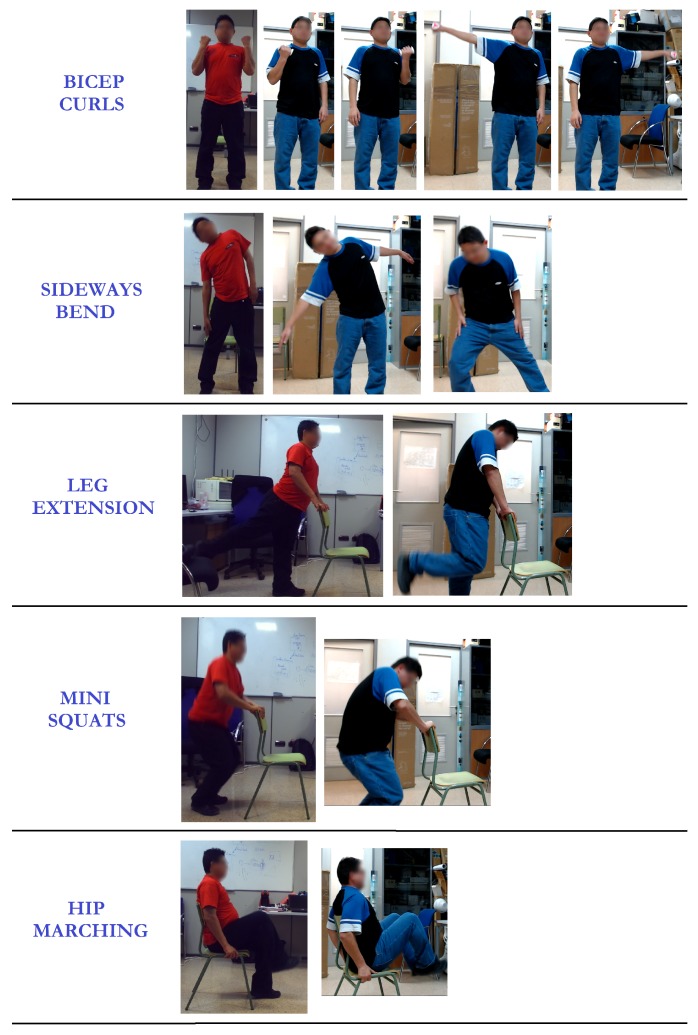
Samples of bad performances for some considered exercises such that the first column indicates the physical exercise to be done; the second column represents the expected pose and the rest of columns illustrate some bad performances of that pose.

However, no error situations were previously considered. Thus, the next step was to define the common errors in an exercise performance to be properly recognised. As illustrated in Figure 5, the number and type of erroneous poses depend on the exercise and the involved limbs. In this way, more precise feedback is provided during the exercise performance and the erroneous poses can be corrected when they take place.

Once the dataset of primitive human poses was defined, the next step was to properly recognise each primitive pose and, from that information, identify and evaluate the physical exercise done. For this, Deep Learning (DL) techniques are used since they provide classifiers that let the system learn data features. Several architectures have been proposed in the literature to address the problem at hand. One of the most successful models for image analysis is the Convolutional Neural Network (CNN). However, CNNs only consider the current input, which makes them inappropriate for action recognition due to its temporal component. As a solution, Recurrent Neural Networks (RNNs) could be used. Unlike CNNs, RNNs make decisions based on both the current and the previous input. Nevertheless, an experimental analysis presented in Reference [44] highlights that a combination of a CNN followed by a RNN provides better accuracy in learning the temporal sequence corresponding to physical exercises than a just RNN architecture.

**Figure 6:**
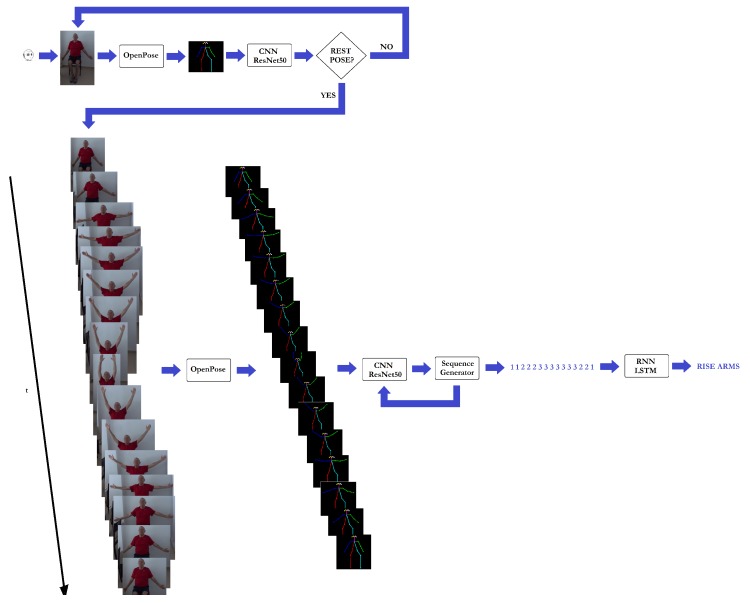
Architecture used for visual exercise recognition.

So, the CNN is in charge of recognising the primitive poses, while the RNN identifies the exercise done from the sequence of pose primitives provided by the CNN. The whole recognition process can be described as follows (see Figure 6): the Pepper robot observes the user until an initial primitive pose is done. That is, each captured image is processed to extract the human skeleton by using Openpose. Note that each limb is coloured differently because of the need to properly distinguish between ambiguous poses like side views. All the detected skeletons are isolated and reshaped to 24×24 skeletons, which are paralleled and introduced into the CNN module to recognise the user’s pose. In particular, a 50-layer Residual Network (*ResNet50* [45]) was used on account of its successful results in visual recognition tasks. When the system detects a human pose starting a physical exercise, two actions can occur. When the recognised pose does not correspond to the initial pose (i.e., rest pose) starting the required exercise, feedback is provided to correct the user’s performance. Otherwise, the monitoring and evaluation stage starts.

So, each captured image is processed so that the human coloured skeleton is obtained. This data is the input to the CNN module (**ResNet50**) to properly recognise the corresponding human pose. This information then goes to the processing module in charge of generating the pose sequence input to the RNN module (i.e., a Long Short Term Memory network (*LSTM* [46]) with 32 units with a dense layer). It is worth noting that the sequence is considered finished when the final exercise of the sequence is done or a rest pose is detected. In addition, the continuous pose analysis allows the system to provide useful feedback at any time, giving the user the opportunity to correct the exercise before finishing it.

## 5. Recommender

The goal of the recommender is to promote active ageing through the suggestion of tailored exercises with the purpose of improving the physical and mental health of older persons. The suggestion of these exercises has two objectives: (1) to improve their strength, balance and flexibility, and (2) to encourage them to lead a more active life, reducing risk factors like depression.

Generally, at a scheduled time, the recommender suggests a personalised set of exercises (one or more) that the user should perform, complying to the minimum of minutes per week of exercises and to the active ageing concept.

The personalised set of exercises is based on the user profile (Figure 1). PHAROS contains several databases that contain the user’s personal information (e.g., name, age, medical condition) and exercise information (e.g., difficulty, appropriateness, repetitions) and the caregiver’s information (e.g., exercises·plans). When suggesting an exercise, PHAROS cross-checks the exercise plan suggested by the caregiver with the user’s personal information (as some plans may be outdated with respect to the user’s current health condition) and thereon enters the selection phase (described in detail below). Currently, following the NHS recommendations, the set of exercises is fixed at 21 unique exercises that are appropriate for older persons. Nonetheless, the recommender is able to deal with an infinite number of exercises, as long as they are in sync with the Physical Exercise Recogniser and are within the user’s physical and cognitive reach.

The previous version of PHAROS used the Glicko2 ranking system [47] to establish the rank of each exercise for each user. In every iteration of the recommender’s execution cycle, a set of exercises was selected and sorted by their ranking (removing previously suggested exercises using a stop-go filter adjusted for two cycles). Thus, if the top ranked exercise was suggested in the past two suggestions, it was removed from the set, with this step being repeated until an unranked exercise was found. The system also took into to account the number of exercises in the set. If they were inferior to 2, this filtering was not applied.

After several tests and validations, it was found that Glicko2 was not appropriate for this usage. In an effort to find a well-tested and balanced ranking system, Glicko2 was chosen over others. The Glicko2 ranking system was designed mainly to be used for board games like chess, where 1v1 is the typical setup. We tried to extrapolate this setup to 1vN, with the following approach: the final selected exercise would be matched against the rest of the exercises (the set of possible exercises) using the Glicko2 algorithm and the ranking of each exercise was updated. The winner/tie/looser title was defined by the performance values given by the *physical exercise recogniser*. Each exercise had a performance value for each user, which was compared against the incoming value related to the suggested exercise and was classified winner/tie/looser if the value was above/same as/below each exercise present in the last set of exercises. Following the algorithm’s behaviour, the winner should increase its rank and the losers decrease their rank.

An issue arose when the algorithm was put to test in large simulation episodes, leading to strange ranking behaviours. Let us consider the following scenario:

- Set of 16 possible exercises (from 21);
- Fixed starting values of 1500 for all exercises;
- Random performance values but over 60%;
- 1000 epochs.

The initial epochs were normal and most of the exercises were selected, but after about 100 epochs, a large deviation was noticed in the exercises that were frequently classified as *losers*. Their rank passed from a stable >1200 points to high negative points (<−5000 points), while the common *winners* showed a stable [1800,2500] points. Furthermore, the stop-go filter was ineffective in overcoming this issue when it occurred. Right from the start, the algorithm showed a preference for the first three exercises that received a high rank, due to the stop-go being configured to only filter the two previous ones, thus creating a cycle of choosing one of these three when they passed the filter.

Due to this issue and the long-term consequences it would cause, we have discarded this algorithm and implemented an alternative one and addressed some proposals presented in our previous publication as future work. In this new version, we have more information about the users and exercises. This feature was improved as an outcome of experiments conducted with older adults (detailed in Section 6). Furthermore, with this update, the new algorithm takes into account the following parameters:

- User performance: how well the user performed each exercise. This feature was also inadvertently improved due to the improvements in the *physical exercise recogniser*.
- Temporal evolution (or *velocity*): if the exercise is in the top suggestions after a period of time, the objective is to boost it and keep suggesting it. This reinforces the performance value (if the user keeps doing it correctly, the exercise is reinforced).
- User likes: if the user lost or gained interest in an activity over time. This is done through post-exercise user questionnaires, as well as through machine learning methods. Studies show that not only injuries or medical problems impact exercise performance but also the attitude towards specific exercises (like/dislike) may influence performance [48]. With this knowledge, the algorithm presents the caregiver with warnings about unexpected performance drops. They can then correlate this issue with relevant injuries or not, and make decisions based on it.
- Batch exercises: as per medical advice, a set of three or less activities are now suggested to the user. This is done to improve the user’s engagement with the exercises (like suggesting a mixture of liked/disliked activities to create a positive response), to achieve a sense of participation in the activity, and to reach the number of recommended minutes per week of exercises.
- Updated internal weights: the weights attributed to each feature can be personalised to each user, translating into direct changes in terms of features’ overall importance.

Thus, the new algorithm now attends to several parameters (not a simple filter and a post-exercise ranking system) and uses the performance as a parameter, replacing the need to re-rank each exercise after each suggestion. We have greatly simplified the algorithm in lieu of pre-algorithm filtering processes (following the batch exercises plan), which adjust the set of available exercises for each suggestion. The selection process is detailed below.

$$E_{User}\subseteq \left\{Exercises\right\}$$

where *E* is a subset of the complete list of exercises for each user. This subset is defined by the caregiver or the medical personnel.

exerciseClassification=L*0.3+V*0.2+P*0.4+A*0.1

where:

L→ user likes;
V→ exercise temporal evolution;
P→ user last performance value;
A→ adjustment value;

As can be observed, four parameters are taken into account in the algorithm. The presented weights are initial and standard and can be tweaked for each user. The reasoning behind these values came from the medical staff.

The *user likes* (*L*) corresponds to the questionnaire completed by the user. This questionnaire accepts a binary response, which is used to reinforce the suggestion procedure. It is not a historic value, meaning that only the last user response is considered.

The *exercise temporal evolution* (*V*) is correlated to the clustering of the number of suggestions of each exercise over small epochs. Currently, the number of epochs is 30. V≥0, for each suggestion made in the last 30 suggestions a value of 1 is added, for each non-suggestion made a value of 1 is subtracted, for each sequential suggestion made, there is an additional value of 0.1. This process is done linearly: as an example, consider the following set of ten suggestions of exercise “*A*”:

$$\begin{array}{c} \{suggested,not-suggested,not-suggested,suggested,not-suggested,\\ not-suggested,suggested,not-suggested,not-suggested,suggested\} \end{array}$$

In this case V=1. Now consider this set:

$$\begin{array}{c} \{suggested,suggested,not-suggested,suggested,not-suggested,\\ not-suggested,suggested,suggested,suggested,not-suggested\} \end{array}$$

In this case V=2.2.

As the name suggests *user last performance value* (*P*) is the user’s performance of that last exercise.

The *adjustment value (A)* is a random binary value used to introduce randomness in the selection of the exercises. Its initial weight is small and does not produce a major change in the sorting mechanism but can be crucial in terms of unravelling a possible tie of exercises.

$$batchExercises=\left\{\left\{x,y,z\right\}:\left\{x,y,z\right\}\subset E_{User}\wedge x\neq y\neq z\vee \left\{y,z\right\}=\left\{\right\}\right\}\vee max_{3}\left(E_{User}\right)$$

where batchExercises is a list of three or fewer exercises belonging to the $E_{User}$. Currently the exercises only follow the rule of not being the same. In the future, we plan to have each exercise come from each group of exercises (of the possible 4) or have predetermined possible combinations of exercises (configured by the caregiver/physician). For instance, exercise E1 may be combined with exercises E4, E7, E8, E11, and E14. Moreover, there is no difference in the number of exercises in a batch, meaning that if a specific suggestion process is configured to have a batch of 2 exercises, all |batchExercises|=2. $max_{3}$ returns the top 3 (or less) exercises.

After this, the exercises are sorted according to their classification and the top ranked one is chosen and suggested to the user.

As explained in previous papers [14], the information is displayed in a *Pepper* robot, along with instructions to the user with pictures and videos. The *Pepper* robot is able to mimic these exercises with the limitation of its hardware, which, being monoped, is only able to perform torso, head and arms-related exercises.

## 6. Experimental Results

With the aim of evaluating our proposal, three experimental scenarios were analysed. Firstly, the recommender system was tested in terms of exercise distribution and frequency in exercise recommendation. Then, an action image dataset was built (tagging all the images by hand based on their pose). This dataset was used to train and evaluate the system accuracy. Finally, the robot assistant was tested in a real environment. Specifically, the pilot study was carried out in a care home located in Alicante (Spain).

### 6.1. Recommender

To test the new algorithm, the problematic scenario (displayed in the beginning of this section) was used to verify the outcome. The parameters were configured in the following way:

- Epochs: 1000;
- *L*: 1 in exercises 1, 5, 7, 12 and 0 on the rest;
- *V*: boosted the last 30 performed exercises;
- *P*: used the last P value. Randomised after each round for the suggested exercises with values [60,100];
- *A*: binary random as per configuration.

**Figure 7:**
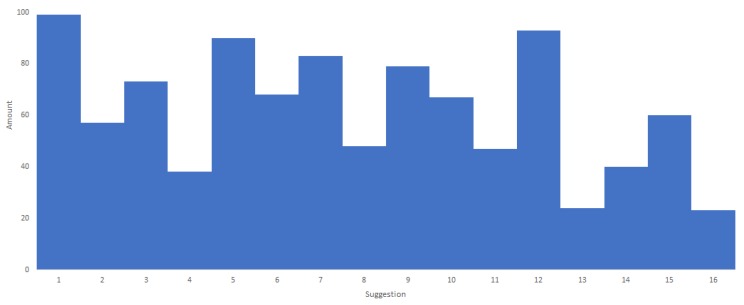
Distribution of the exercises using the new algorithm applied to the problematic scenario.

Figure 7 shows the frequency distribution of the exercises. As can be observed, the frequency of the suggested exercises is not evenly distributed and shows preference for some exercises over others but the difference is not heavily skewed to only a short set of exercises. This result is optimal to the environment where it operates.

**Figure 8:**
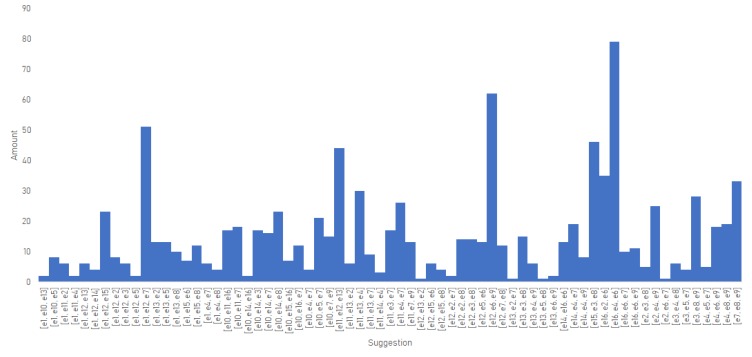
Distribution of a set of three exercises per suggestion using the new algorithm applied to the problematic scenario.

Figure 8 shows the same scenario but, in this case, suggesting three exercises. Remembering the algorithm, the exercises cannot be repeated nor is their order important, thus {e1,e2,e1} is not allowed and {a1,a2,a3}={a2,a1,a3}. In this scenario, it is observable that yet again there is a preference for certain sets of exercises (in this case due to the temporal evolution and performance) but it is not severely skewed like the old algorithm.

In senior residences and at users’ homes, several parameters can change (nudges from the caregivers or other factors), which may lead to a different result from what we propose. What we try to demonstrate is that by itself the recommender is not overly reactive and skewed and even if there are no nudges over time it is able to make balanced suggestions.

### 6.2. Early Evaluation of the Exercise Recogniser

In the early stage, staff at university were recruited to do all the exercises. A total of twelve persons aged between 25 and 65 years were recorded (in laboratory and at-home environments) while they repeated each exercise 5 times. In addition, they were asked to also engage in the most typical erroneous poses considered in this work. This data was used to create an action dataset and to validate our proposal. Thus, a total of 58,430 images distributed among 42 poses and 30 physical exercises, were divided into 70% for training, 15% for test and 15% for validation. It is worth noting that the images used for validation correspond to two people not included in the other subsets, that is, they were completely new to the system. Under these settings, the system achieved an accuracy of 93.98% for training, 91.37% for test and 90.70% for validation with a 250-epoch running. Despite the good performance, there is some confusion when the exercise is performed towards one side or another, as illustrated in Table 1. The reason lies in the similarity between the pose sequences of the exercises. In particular, there is more confusion between sequences composed of 3 human poses (e.g., *sideways bend left and right*) than those with 5 human poses (e.g., *neck stretch left and right*). In addition, although the left and right side are differently coloured, there are still some classification errors in the CNN part, which also leads to these confusions in exercise recognition.

### 6.3. The Pilot Study

As mentioned above, the robot assistant was tested in a real scenario. Specifically, the pilot study was carried out at the Doña Rosa care home, located in Alicante (Spain), where there are more than 70 residents aged between 60 and 90 years. These individuals live in the residence full time, so their basic activities (food, medical assistance, etc.) must be taken care of. The Doña Rosa residents present different levels of disability; certain physical impairments or others types of disabilities. However, most are physically valid. For these individuals, an active physical program could improve their daily life.

**Figure 9:**
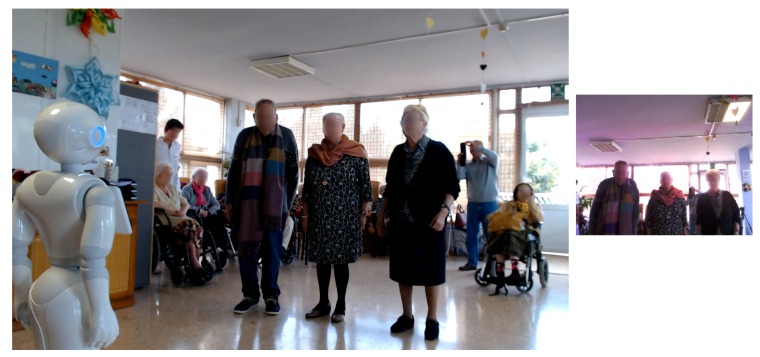
The left image shows the set-up of the pilot study where the robot is located in front of the users and there are some observers behind them; while the right image corresponds to the robot view.

We designed a pilot program with a total of eight participants (see Figure 9), who were selected from among those with most capabilities. First, the robot was introduced, using some entertaining shows: the robot sang and danced old fashion songs, told popular stories and reacted to the touch of the people in the residence. This was done to create a more supportive environment. Then, the eight participants in the study were divided into three groups (3, 3 and 2 persons). For each group, the robot indicated the exercise to be done, imitating the exercise. At the same time, it captured the movement from each person while monitoring them.

The overall performance of PHAROS was thus analysed. One key issue was the multi-person exercise recognition since all the training and tests were performed for one person individually. Therefore, given that group sessions took place, each captured image resulted in several coloured skeletons analysed in parallel execution. This performance highlighted that the varying number of people within the scene did not have any significant effect on the execution time, obtaining a real-time performance during the entire pilot study. However, there were some some conflicts in providing feedback during the exercise. That is, the feedback was provided based on the recogniser response. However, this feedback should be provided based on the criticality of the error in terms of its effect on user’s health.

On the other hand, the usability of PHAROS was analysed by means of a system usability scale (SUS) questionnaire. This questionnaire was composed of the following 7 items:

- You liked the activity
- The activity was funny
- You found the activity demanding
- You got bored
- The activity encouraged you
- The activity was collaborative
- You would do it again

**Figure 10:**
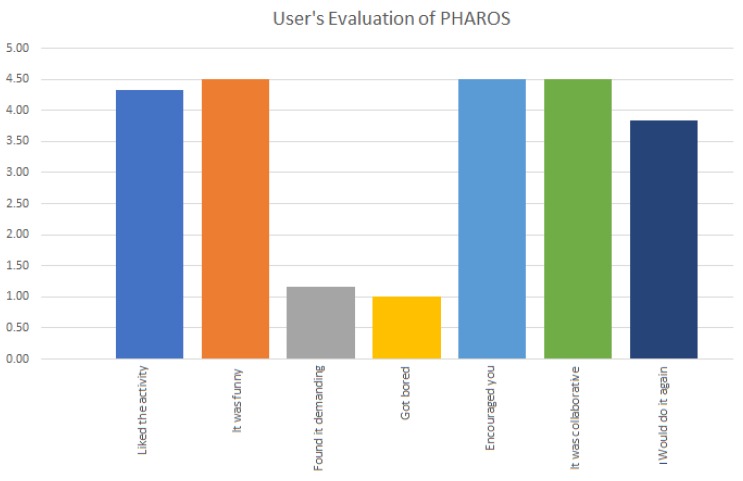
User evaluation of PHAROS on a five-point Likert scale.

A five-point Likert scale was used to measure the participant’s opinion and attitude towards this system. The possible choices were: very unsatisfied (1), unsatisfied (2), neutral (3), satisfied (4) and very satisfied (5). As illustrated in Figure 10, the average score obtained for each item highlights the user satisfaction with the system. Note that the demand of the proposed exercises was evaluated as low since they are exercises included in their daily sessions. This is an important issue, since PHAROS is designed to work without therapist supervision and its ease and understanding are key issues.

## 7. Conclusions

The change in the global economic model, together with population ageing, has led to an ever-increasing need for healthcare services. As a response, technological solutions are being developed. This is the case of our previous work, PHAROS, an assistive robot for promoting and monitoring physical activity. In this paper, we have presented an evolved version of this system, PHAROS 2.0. So, the action dataset has been extended to include bad exercise performances. This improvement has allowed the system to provide more precise feedback during the exercise (correcting the user’s pose when necessary) and, consequently, providing more accurate information to the medical personnel about the user’s performance in each session. In addition, a new neural network architecture has been implemented. In this case, a *ResNet50* together with a *LSTM* have been used. So, the system’s accuracy is almost 91% (i.e., 90.7%) with a 250-epoch training. Note that most of the misclassifications result from the bad distinction in body-rotation exercises. Therefore, as future work, we plan to work on a different human skeleton code overcoming this issue.

This detailed data feeds the recommender, what is able to better fit the daily workout to the user’s needs based on the specialist recommendations. Several experiments were carried out with the aim of evaluating the effect of these improvements in the functioning of the system. These results highlight the stability of the system, recommending physical exercises according to the user needs and abilities in an engaging way.

Finally, the whole system was validated by means of a pilot study in a senior care home located in Alicante. For that, eight people with ages between 60 and 90 years participated in this study. These participants were divided into three groups of 3, 3 and 2 people, respectively. The monitoring tests validate the PHAROS performance. Additionally, an SUS study revealed the great acceptance of the users as well as its utility as an exercise promoter.

Despite the advances, there is still some work to be done. Thus, as future work, three different research lines are proposed. Firstly, a graphical interface presenting the information to the medical personnel is advisable. In addition, the wide range of erroneous poses when an exercise is done, highlights the need to search for a more flexible way to deal with them. The feedback module must also be improved to play a role more similar that of the therapist, enriching the user’s experience. 

## Figures and Tables

**Table 1:**
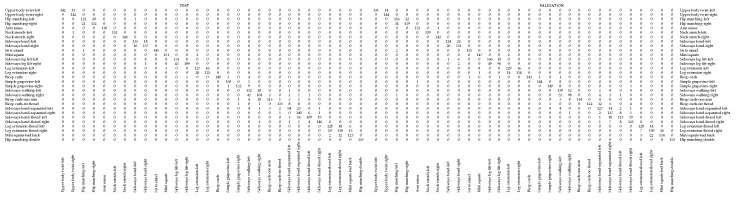
Confusion matrix for test and validation data with 250 epochs.
